# FZD2 regulates cell proliferation and invasion in tongue squamous cell carcinoma

**DOI:** 10.7150/ijbs.33881

**Published:** 2019-08-24

**Authors:** Li Huang, Er-Ling Luo, Jing Xie, Rui-Huan Gan, Lin-Can Ding, Bo-Hua Su, Yong Zhao, Li-Song Lin, Da-Li Zheng, You-Guang Lu

**Affiliations:** 1 Department of Dentistry, The First Affiliated Hospital of Fujian Medical University, 20 Chazhong Road, Fuzhou, 350005, China; 2 Department of Preventive Dentistry, School and Hospital of Stomatology, Fujian Medical University, 246 Yangqiao Middle Road, Fuzhou 350000, China; 3 Key Laboratory of Ministry of Education for Gastrointestinal Cancer, Fujian Medical University, 1 Xue Yuan Road, University Town, Fuzhou 350122, China; 4 Department of Pathology, School and Hospital of Stomatology, Fujian Medical University, 246 Yang Qiao Middle Road, Fuzhou 350000, China; 5 Key laboratory of Stomatology of Fujian Province, School and Hospital of Stomatology, Fujian Medical University, 88 Jiao Tong Road, Fuzhou 350004, China

**Keywords:** FZD2, Tongue cancer, Proliferation, WNT signaling pathway

## Abstract

Many studies have shown that FZD2 is significantly associated with tumor development and tumor metastasis. The purpose of the present study was to gain insight into the role of FZD2 in the cell proliferation and invasion of tongue squamous cell carcinoma. According to TCGA-HNSC dataset, among the 10 Frizzled receptors, FZD2 exhibited the highest degree of differential expression between cancer tissues and normal tissues, and the overall survival of patients with higher FZD2 levels was shown to be significantly shorter compared with those with lower FZD2 levels. The upregulation of FZD2 in clinical tongue cancer tissues was validated by real-time PCR. Knockdown of FZD2 inhibited the proliferation, migration and invasion of CAL-27 and TCA-8113 cells, whereas overexpression of FZD2 led to the opposite results. Further analysis revealed that FZD2 is positively correlated with WNT3A, WNT5B, WNT7A and WNT2 and is negatively correlated with WNT4. These results indicated that FZD2 may act as an oncogene in tongue squamous cell carcinoma. Therefore, FZD2 may be a target for the diagnosis, prognosis and gene therapy of tongue cancer.

## Introduction

Oral cavity cancers are common malignant tumors, and the most common oral cavity tumors are oral and maxillofacial malignant tumors. These tumors have a high malignancy, invasive growth, and early regional lymph node metastasis, especially tongue squamous cell carcinoma 1. It is reported that there are approximately 500,000 new cases of tongue cancer every year worldwide, and the incidence of tongue cancer is increasing each year with a younger age of onset throughout the world 2. The tongue has numerous lymphatics and abundant blood circulation, and there are frequent movements of the tongue; these factors promote the early transfer of cancer cells to the adjacent tissues and organs, such as the lymph nodes, floor of the mouth, throat and neck. Moreover, although the treatments for tongue cancer are constantly improving with the advancement of technology, the five-year survival rate is still not ideal; for patients with metastasis, the five-year survival rate is lower 3. Therefore, it is important to identify possible molecular targets that are relevant to development of tongue cancer. Studying the corresponding molecular mechanisms will introduce a new experimental foundation and provide possible targets for the early diagnosis, new treatment strategies and prognostic analysis of tongue cancer 4.

The WNT signaling pathway is a relatively conservative signaling pathway throughout evolution with a wide range of biological effects, including embryonic development 5, organ formation, stem cell renewal, cell survival, apoptosis and necrosis. This pathway regulates most biological phenomena in living organisms 6. The primary members of the signaling pathway include a secreted protein (Wnt), transmembrane receptors (FZD1-10), β-catenin, glycogen synthase kinase 3β (GSK3β) and others. As a receptor in the WNT signaling pathway, FZD2 is a highly conserved signaling molecule that belongs to the G protein coupled receptor family 7. The FZD genes encode for seven transmembrane proteins, whose amino terminus is located extracellularly with a cysteine-rich domain, which is the binding domain with a high affinity for Wnt proteins 8-9. In the process of signal transduction, when the extracellular secreted protein Wnt binds to the transmembrane receptor FZD, the expression of downstream genes can be activated by the β-catenin-dependent pathway 10 or the β-catenin-independent pathway 11.

Many studies have shown that the abnormal expression of FZD2 is significantly associated with the development of many tumors, such as gastric cancer 12, hepatocellular carcinoma 13 and endometrial cancer 14, in which FZD2 plays oncogenic roles. However, FZD2 has also been reported to be a tumor suppressor gene in salivary adenoid cystic carcinoma 4. FZD2 plays different roles in different tumors; even in the same tumor, the role of FZD2 can vary due to the different microenvironments within the tumor. Although some studies have speculated that FZD2 may contribute to carcinogenesis in oral squamous cell carcinoma cell lines 15-16, the roles of FZD2 in tongue squamous cell carcinoma remain ambiguous.

In this study, we aimed to illuminate the roles of FZD2 in tongue squamous cell carcinoma. We analyzed the differential expression levels of FZD2 between clinical tongue cancer tissues and the adjacent tissues. Then, we silenced and overexpressed FZD2 in tongue squamous cell carcinoma cells to investigate changes in cell proliferation, migration, and invasion *in vitro* and in a nude mice xenograft tumor model. Furthermore, we also compared the expression of FZD2 between parental cells and highly metastatic cells of tongue squamous cell carcinoma.

## Results

### FZD2 is overexpressed in head and neck squamous cell carcinoma and tongue cancer

**Figure 1:**
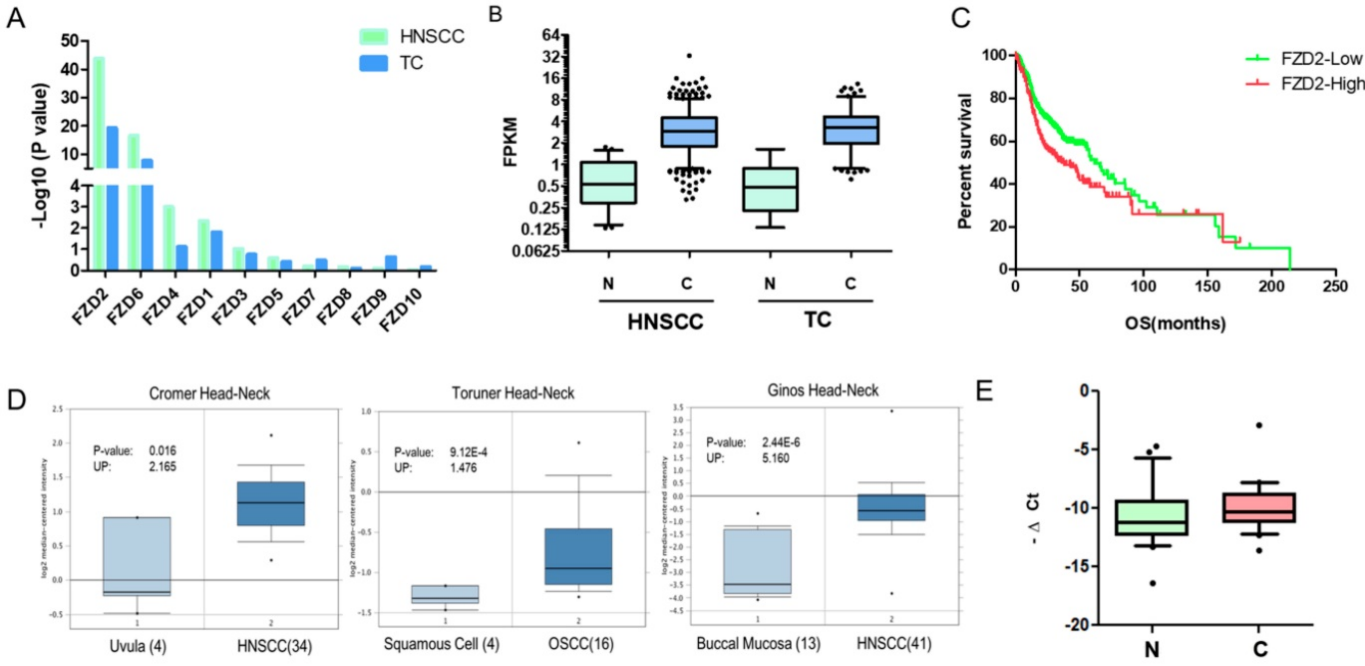
** FZD2 is overexpressed in head and neck squamous cell carcinoma**. The differential expression of Frizzled receptors in head and neck squamous cell carcinoma 501 cases of cancer and 41 cases of normal) and tongue cancer (149 cases of cancer and 15 cases of normal) from the TCGA database and the -Log10(P value) by t test was showed (A). The expression of FZD2 in head and neck squamous cell carcinoma and tongue cancer compared with normal tissues (B, N: normal tissues, C: cancer tissues). The overall survival of patients with HNSCC according to the different expression levels of FZD2 based on the TCGA database (C, 194 cases with high and 307 cases with low FZD2 expression, P=0.02 by Mantel-Cox test). Representative images of the upregulation of FZD2 in HNSCC from the Oncomine database (D). The expression of FZD2 in 44 pairs of tongue cancer tissues and adjacent tissues was detected by real-time PCR (E, P<0.05 by t test, n=44).

Since the Frizzled receptor has an indispensable position in the Wnt signaling pathway, we aimed to investigate the role of FZD in the development of tongue cancer. In this study, we first explored the expression of Frizzled receptors in head and neck squamous cell carcinoma and tongue cancer from the TCGA database (https://gdc.cancer.gov). According to this publicly available database, among the 10 FZD genes, the differential expression of FZD2 in head and neck squamous cell carcinoma (501 cases of cancer and 41 cases of normal) and tongue cancer (149 cases of cancer and 15 cases of normal) was the most obvious compared with normal tissues (Fig 1A). We further analyzed the data and determined that FZD2 was significantly increased in head and neck squamous cell carcinoma and tongue cancer compared with normal tissues (Fig 1B); moreover, the overall survival of patients with head and neck squamous carcinoma with high FZD2 expression (n=194, FPKM>means) was significantly decreased (Fig 1C, P=0.02) when compared FZD2 low expression (n=307, FPKM<means). We also analyzed the expression of FZD2 in the Oncomine database using unbiased bioinformatics (http://www.oncomine.org), and we found that FZD2 is upregulated in 10 head and neck cancer datasets and is not downregulated in any dataset when the threshold was set as P=0.05, Fold Change=1.5, and Fig 1D shows some representative images of FZD2 overexpression. Based on the results of this data mining, FZD2 may play an oncogenic role in HNSCC.

To confirm the relationship between FZD2 and tongue cancer, we used real-time PCR to detect the expression of FZD2 in 44 pairs of tongue cancer tissues and their corresponding adjacent tissues. The results showed that the expression level of FZD2 in tongue cancer tissues was higher than in the corresponding adjacent tissues (Fig 1E, P<0.05). Further analysis of clinical information revealed that FZD2 expression was higher in moderately or poorly differentiation group than that in well differentiation tissues (Table 1, P<0.05). Collectively, these data suggest that FZD2 may contribute to carcinogenesis in tongue cancer.

### FZD2 promotes the proliferation of tongue squamous cell carcinoma cells *in vitro*

**Figure 2:**
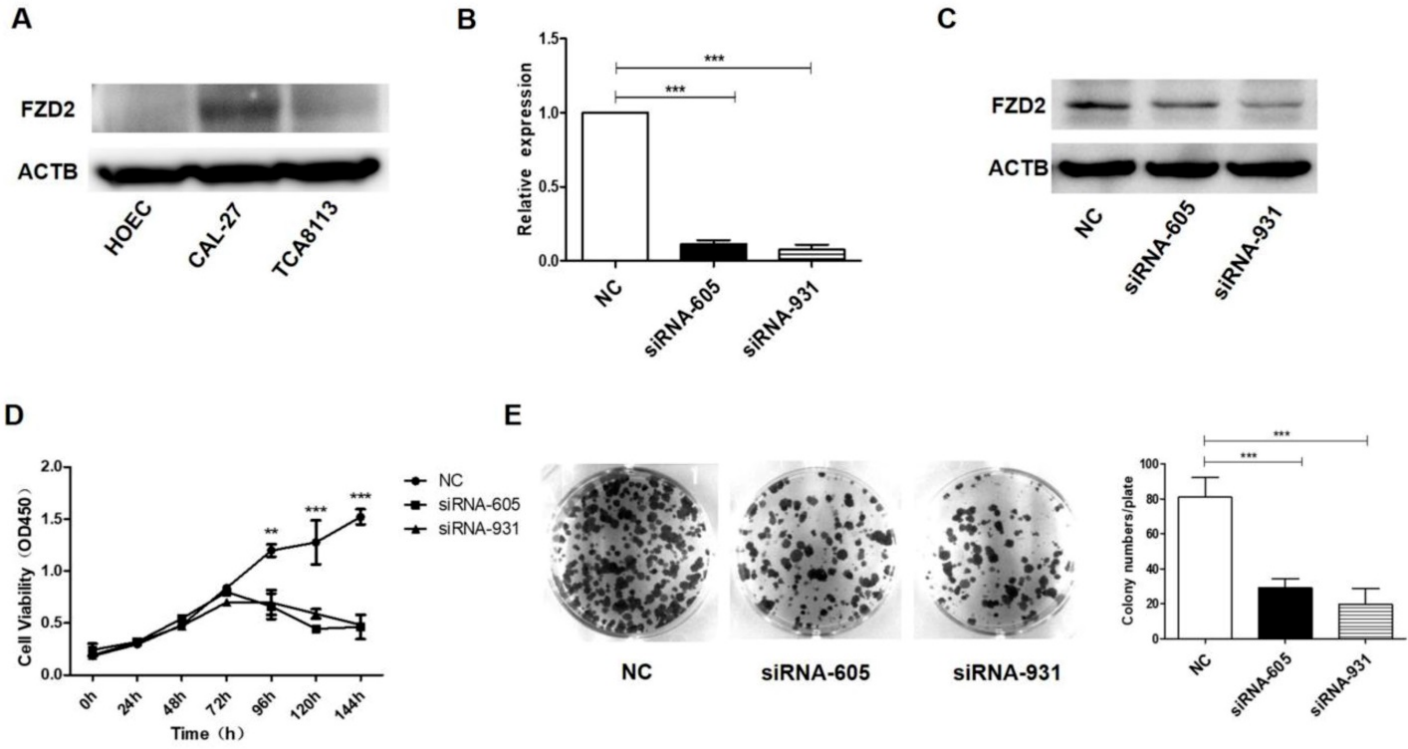
** The siRNA-mediated knockdown of FZD2 inhibited the growth of CAL-27 cells *in vitro*.** The expression of FZD2 in tongue cancer cell lines CAL-27 and TCA-8113 and normal human oral epithelial cell line HOEC was detected by Western blot (A). After siRNA transfection in CAL-27 cells, the expression of FZD2 was measured by real-time PCR (B) and Western blotting (C), the cell proliferation was detected by CCK8 assay (C, P < 0.01 by One-Way ANOVA followed by Tukey's multiple Comparison test from 96 h to 144 h) and colony-formation assay (D, P<0.005 by One-Way ANOVA followed by Tukey's multiple Comparison test, n=3).

To confirm the functional effects of FZD2 on the proliferation of cancer cells, the expression of FZD2 in tongue cancer cell lines CAL-27 and TCA-8113 and normal human oral epithelial cell line HOEC was detected by Western blot. As showed in Fig 2A, the FZD2 level was highest in CAL-27, and low in TCA-8113 and almost negative in HOEC cells. FZD2-targeting siRNAs and the negative control (NC) were transiently transfected into CAL-27 and TCA-8113 cells. The mRNA and protein expression levels of FZD2 were detected by real-time PCR (Fig 2B and Fig S1A) and western blotting (Fig 2B and Fig S1A), respectively. As expected, the expression of FZD2 was efficiently reduced in the siRNA-605 and siRNA-931 groups compared with the negative control group. The CCK8 (Fig 2C and Fig S1B) and colony formation assays (Fig 2D and Fig S1C) indicated that the siRNA-mediated knockdown of FZD2 significantly inhibited the growth of tongue cancer cells. In addition, silencing FZD2 expression inhibited CAL-27 cells proliferation.

**Figure 3:**
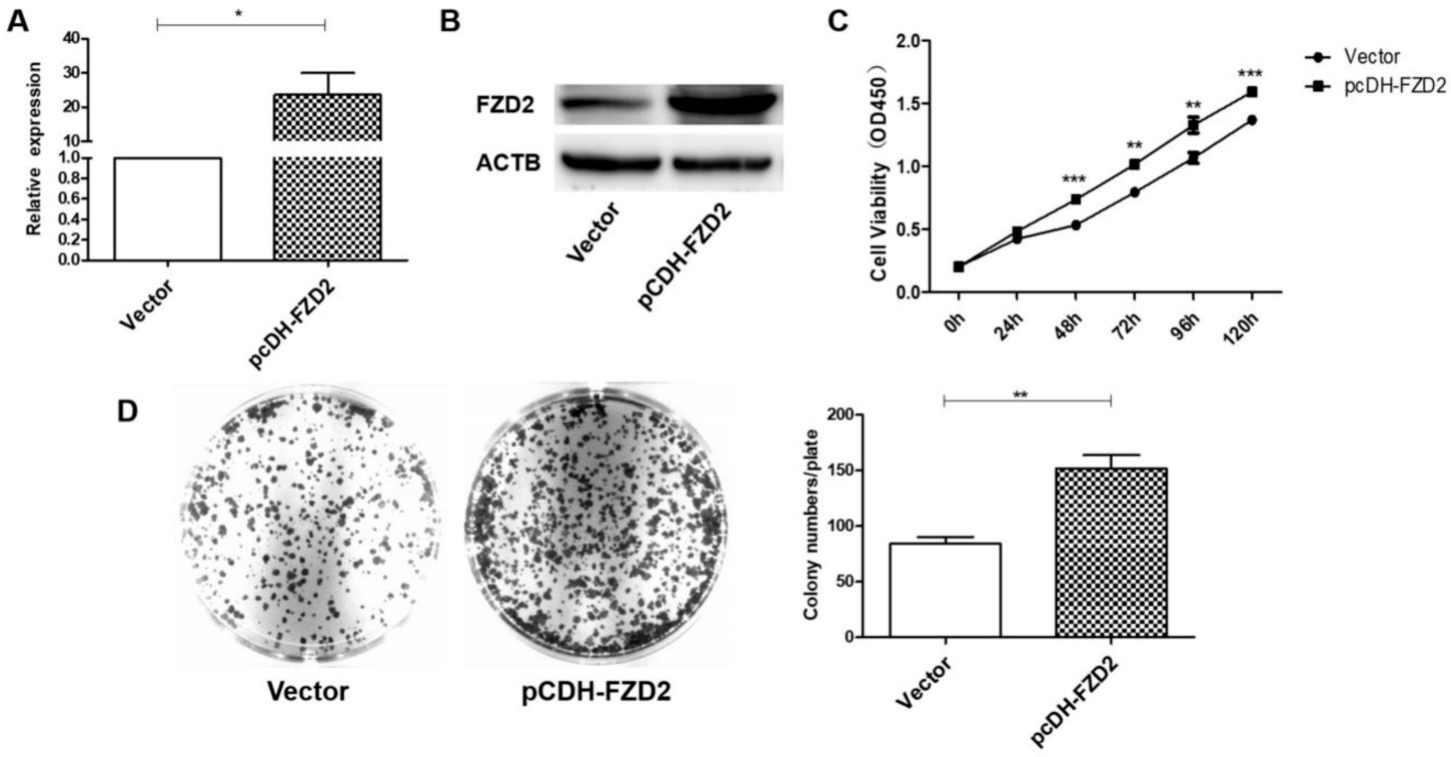
** Upregulation of FZD2 in CAL-27 cells promoted cells proliferation *in vitro*.** After transfected with plasmid, the expression of FZD2 was measured by real-time PCR (A) and Western blotting (B), the cell proliferation was detected by CCK8 assay (C, P < 0.01 by One-Way ANOVA followed by Tukey's multiple Comparison test from 48 h to 120 h) and colony-formation assay (D, P<0.01 by One-Way ANOVA followed by Tukey's multiple Comparison test, n=3).

To further verify the contribution of FZD2 to the proliferation of cancer cells, a plasmid carrying the correct coding sequence of the intracellular cytoplasmic domain of FZD2 (pCDH-FZD2) and the corresponding negative control plasmid (Vector) were generated and transfected into CAL-27 and TCA-8113 cells. The results of real-time PCR (Fig 3A and Fig S2A) and western blotting (Fig 3B and Fig S2B) showed that the expression of FZD2 was elevated in the CAL-27 and TCA-8113 cells. The overexpression of FZD2 in the tongue cancer cells clearly enhanced short-term cell growth as measured by the CCK8 assay (Fig 3C and Fig S2C). Furthermore, the colony formation assay (Fig 3D and Fig S2D) demonstrated that the single cell proliferation was increased after transfection with the plasmid (pCDH-FZD2). Taken together, these results validate the finding that FZD2 promotes proliferation of tongue cancer cells *in vitro*.

### Knockdown of FZD2 represses tumorigenicity *in vivo*

**Figure 4:**
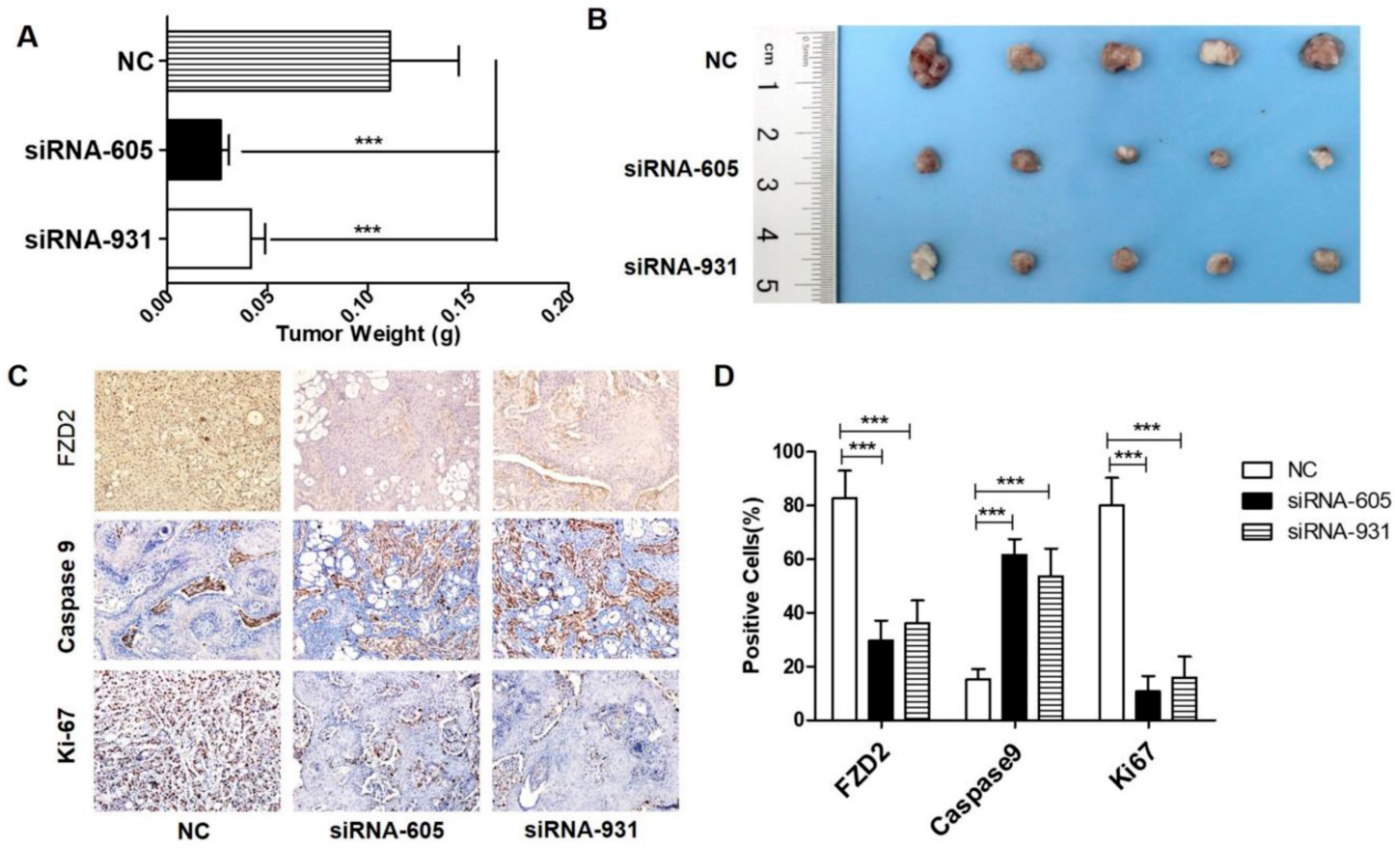
** Knockdown of FZD2 represses tumorigenicity* in vivo.*** After siRNA transfection in CAL-27 cells, the cells were injected into the flanks of nude mice, the tumor-bearing animals was were sacrificed in experimental observation for 28 days and the tumors were removed for weighing (A, P<0.001 by One-Way ANOVA followed by Tukey's multiple Comparison test) and photographing (B). The expression of FZD2 (upper panel), cleaved Caspase-9 (middle panel) and Ki-67 (lower panel) in the xenograft tumors were detected by immunohistochemistry (C) (DAB, 200 x) and the different groups of positive cells were counted and compared(D, P<0.001 by One-Way ANOVA followed by Tukey's multiple Comparison test).

Our *in vitro* studies confirmed that the downregulation of FZD2 expression effectively inhibits the proliferation of tongue cancer cells. To further investigate the effect of FZD2 on tumorigenicity *in vivo*, CAL-27 cells that were separately transfected with different siRNAs (NC, siRNA-605 and siRNA-931) were injected into the flanks of nude mice. During the experimental observation period of 28 days, the weight of nude mice was measured every 3 days after the injection, and the length and diameter of the tumors were measured. As seen from the tumor weight (Fig 4A) and tumor sizes (Fig 4B), the tumor growth rate of the siRNA-605 group and siRNA-931 group was significantly lower than that of NC group. Furthermore, the FZD2 protein level and proliferation index (Ki-67) of xenograft tumors of the siRNA transfection group were decreased compared with the negative control, while the apoptotic cells (as indicated as Caspase-9 level) were increased (Fig 4C, P<0.001). Taken together, these data suggest that the downregulation of FZD2 expression in tongue squamous cell carcinoma cells effectively inhibits the formation and growth of tongue cancer *in vivo*.

### FZD2 increases cell migration and invasion *in vitro*

**Figure 5:**
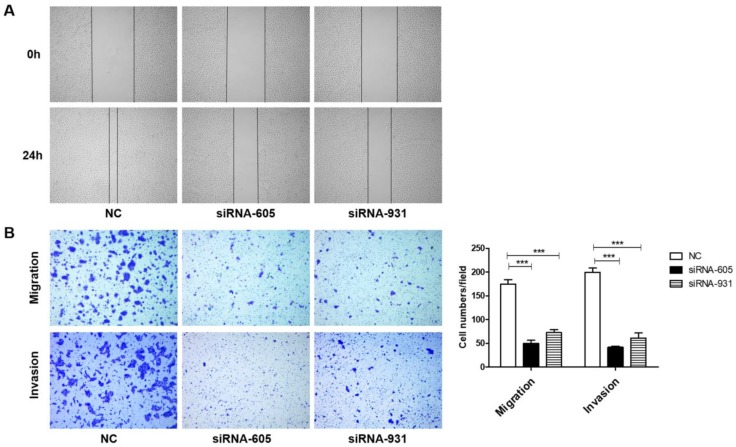
** Downregulation of FZD2 suppressed the migration and invasion of CAL-27 cells *in vitro***. After inhibition the expression of FZD2, the cells migration and invasion were detected by wound healing assay (A) and transwell assay coated with or without matrigel (B, P<0.005 by One-Way ANOVA followed by Tukey's multiple Comparison test), the representative images of transwell inserts coated without (upper panel) or with (lower panel) matrigel.

**Figure 6:**
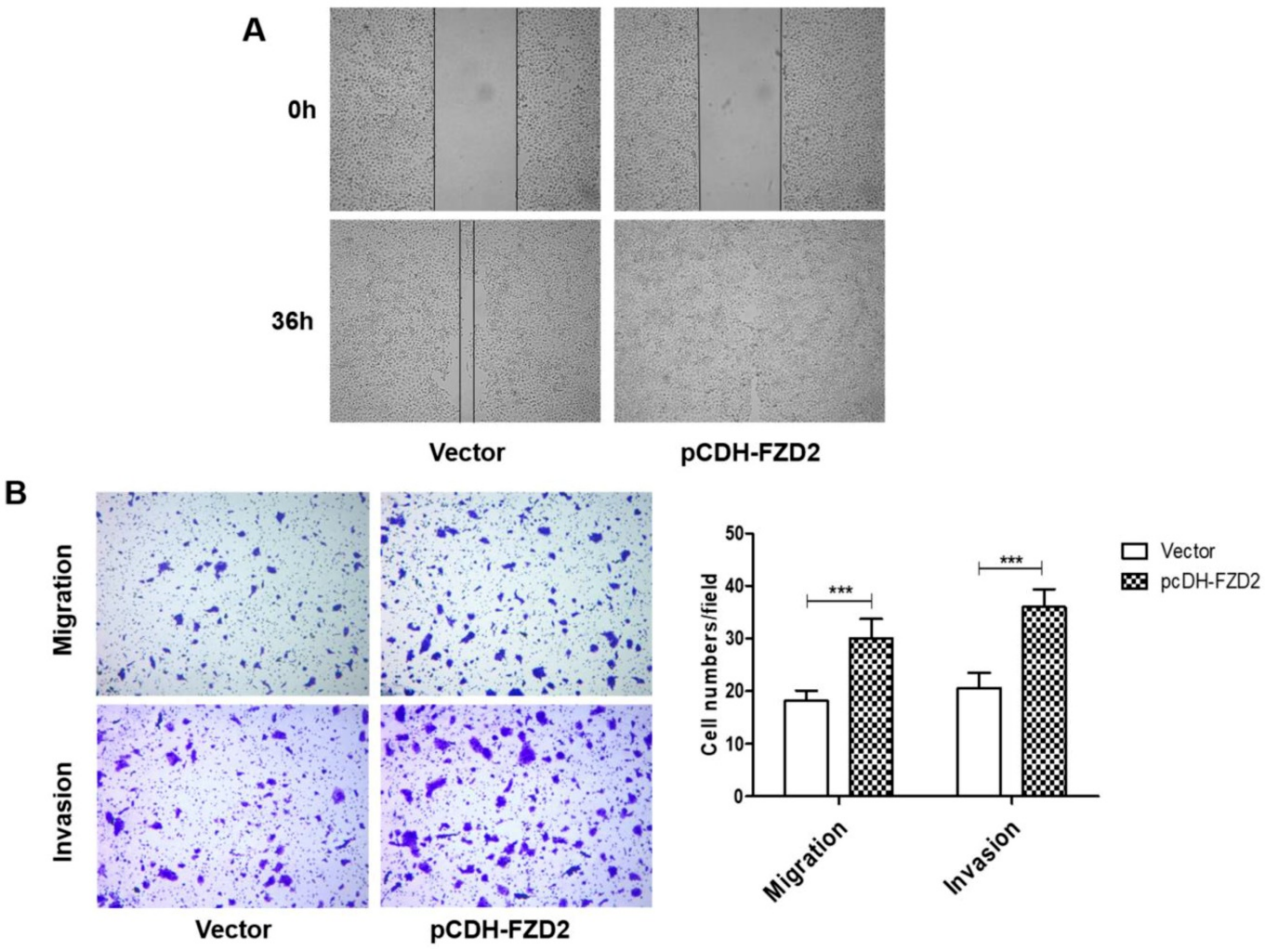
** Upregulation of FZD2 in CAL-27 cells promoted cells migration and invasion *in vitro***. After transfected with plasmid, the cell motility and invasiveness were boosted, as detected by wound healing (A) and transwell assays (B, P<0.005 by One-Way ANOVA followed by Tukey's multiple Comparison test), the representative images of transwell inserts coated without (upper panel) or with (lower panel) matrigel.

We next investigated whether FZD2 could promote the migration and invasion of tongue squamous cell carcinoma cells. The results of the wound-healing assay (Fig 5A) showed that, 24 h after scratching, most of the blank areas of the negative control were covered by cells, whereas the siRNA-transfected groups still had wide blank areas. The transwell assay (Fig 5B and Fig S1D), which used transwell inserts that were uncoated or were coated Matrigel, demonstrated that the invasion and migration of tongue cancer cells were decreased in the siRNA-605 group and siRNA-931 group compared to the NC group. Inversely, when FZD2 was overexpressed in CAL-27 and TCA-8113 cells, the cell motility and invasiveness were boosted, as detected by wound healing (Fig 6A) and transwell assays (Fig 6B and Fig S2E). The results revealed that FZD2 plays an oncogenic role in tongue cancer and contributes to the migration and invasion of tongue squamous cell carcinoma cells.

### Correlation between FZD2 receptor and Wnt ligands

It has been reported that a single Wnt ligand can bind to several FZD receptors, whereas one FZD receptor may interact with multiple Wnt ligands 18. To further investigate the effects of the FZD2 receptor binding to different WNT ligands in head and neck squamous carcinoma, we analyzed the coexpression of the FZD2 gene with 18 WNT ligands in the TCGA-HNSC dataset to systematically map FZD2-Wnt interactions in tongue cancer. The results revealed that FZD2 is positively correlated with WNT3A, WNT5B, WNT7A and WNT2 and is negatively correlated with WNT4 (Fig 6A). When the 501 samples were divided into two groups according to the expression level of FZD2, the expression of WNT3A, WNT5B and WNT2 was significantly higher and that of WNT4 was significantly lower (Fig 6B) in the FZD2-high group (n=194) compared with the FZD2-low group (n=307). These results suggest FZD2 may serve as a receptor for WNT3A, WNT5B and WNT7A.

## Discussion

Due to the special anatomy and function of the tongue, tongue cancer has early metastasis and a high rate of relapse after surgery. In the past few decades, great progress has been achieved in the treatment of tongue cancer, but the 5-year survival rate of patients with tongue cancer has been maintained at 40% to 50% 19. Moreover, patients with local or distant metastases have a poor prognosis, and the long-term survival conditions have not improved significantly 20. Strengthening the understanding of the molecular mechanisms of FZD2 carcinogenesis can improve the prognosis and quality of life of patients.

The WNT/Frizzled signaling pathway regulates cell growth, differentiation, apoptosis and self-renewal. This pathway is an evolutionarily conserved signaling pathway that not only plays a role in embryonic development and certain normal physiological processes but also mediates the development of various tumors through the β-catenin-dependent pathway or the β-catenin-independent pathway 21-22. Some studies have shown that the receptor gene FZD2 is abnormally overexpressed in many tumors 23-24. Asano T et al. 25 demonstrated that FZD2 is highly expressed in hepatocellular cancer tissues compared to adjacent tissues and that patients with a mesenchymal tumor in which FZD2 expression was significantly higher than in the epithelial tumor were more prone to experiencing earlier recurrence compared with those with an epithelial tumor. Liu et al. 26 analyzed 62 cases of esophageal squamous cell carcinoma tissue samples by real-time PCR and determined that the expression of Cyclin D1, C-MYC, MMP2 and FZD2 was significantly upregulated in the cancer tissues. In addition, Rhee CS et al. 16 utilized real-time PCR analysis to demonstrate that FZD2 was overexpressed in head and neck SCC cancer cell lines compared to normal bronchial epithelial cells or primary oral squamous epithelial cells. In our results, according to the TCGA and Oncomine databases, FZD2 is highly expressed in head and neck squamous cell carcinoma and is closely related to the survival of patients. In addition, our results demonstrate that the expression of FZD2 in tongue squamous cell carcinoma is higher than in the corresponding peritumoral tissue. Therefore, we hypothesize that FZD2 may contribute to carcinogenesis in tongue cancer.

When the WNT/Frizzled signaling pathway is abnormally expressed, tumor proliferation, invasion, immunity, chemoresistance and stemness are frequently dysregulated 27. A previous study showed that the binding of the FZD2 receptor to WNT3A and WNT5A promotes tumor proliferation, migration and invasion through activating the WNT-β-catenin signaling pathway and the β-catenin-independent pathway in neuroblastoma 28. Qi et al. 29 explored the function of miR-30a-3p/5p in esophageal squamous cell carcinoma and determined that the downregulation of miR-30a-3p/5p promotes esophageal squamous cell carcinoma cell proliferation by activating the expression of WNT2 and FZD2. To further validate the carcinogenic effect of FZD2 in tongue cancer, we observed the changes in cell proliferation and migration *in vitro* and *in vivo* after altering the expression of FZD2 in tongue squamous cell carcinoma cells. The CCK-8 assay, colony-formation assay and subcutaneous xenograft models revealed that cell proliferation was suppressed following the siRNA-mediated downregulation of FZD2 expression. Conversely, the overexpression of FZD2 promoted cell growth. Other studies have also shown the effect of FZD2 on promoting proliferation. Tomizawa et al. 12 reported that FZD2-targeting shRNA effectively inhibited cell proliferation in gastric cancer, and the same group also observed a decline in cyclin D1 levels following the downregulation of FZD2 expression. Another study showed that FZD2 was expressed in tumorous HCC tissue but not in the surrounding nontumorous tissue and that the proliferative capacity of hepatocellular carcinoma cells was decreased after transfection with FZD2-targeting shRNA. Therefore, the authors speculated that FZD2 may be an ideal molecular therapeutic target for treating HCC 30.

Tumor metastasis is a complex biological process that involves the detachment of tumor cells from primary tumors, followed by migration and colonization in distant organs, which is the primary cause leading to failure of tumor therapy and even patient death 31. Our results showed that FZD2 enhanced cell invasion and migration in CAL-27 cells following the overexpression of FZD2; in contrast, invasion and migration were reduced following the inhibition of FZD2. It has been reported that the activation of the WNT5A/FZD2-induced noncanonical WNT pathway (NCWP) promotes the progression of advanced and metastatic prostate cancer and induces the epithelial-to-mesenchymal transition (EMT) in certain cancers 32. Based on our analysis of the FZD2-WNT correlation, we speculate that FZD2 combined with different Wnt ligands will have different effects on the development of tongue cancer. An earlier study revealed that FZD2 promotes cell motility and invasiveness in oral squamous cell carcinoma cells via the regulation of the STAT3 pathway 15. Bian et al. 14 reported that overexpression of FZD2 increases cell migration and induces an EMT phenotype by activating canonical WNT signaling in endometrial cancer. Furthermore, FZD2 was considered likely to be a potential biomarker for tumor metastasis and a target for future therapies for this disease. Additionally, Gujral and colleagues 33 showed that FZD2 drives the EMT and cell migration in colorectal cancer by activating the Fyn and STAT3 signaling pathways. The authors asserted that FZD2 may serve as a gene signature to predict tumor metastasis and overall survival in patients. Overall, the abovementioned studies provide a feasible direction for future in-depth studies on the molecular mechanisms underlying FZD2 in tongue cancer.

In conclusion, we conclude that FZD2 acts as an oncogene in tongue cancer, promoting cell growth, invasion and migration. These findings may provide a new strategy for gene-targeted therapy in the treatment of tongue cancer through suppressing the expression of FZD2.

## Materials and methods

### Clinical samples

Tissue samples were obtained from the First Affiliated Hospital of Fujian Medical University. A total of 44 cases of tongue carcinoma tissues and the corresponding adjacent normal tissues were included. This study was approved by the Institutional Review Board of Fujian Medical University, and written informed consent was obtained from each participant.

### TCGA datamining

The mRNA expression data and clinical information from the HNSC dataset were downloaded from the TCGA data portal (http://gdc.cancer.gov). The dataset was obtained on July 28, 2018, which included 501 HNSC samples and 41 normal tissues. Among these samples, there are 149 tongue cancer samples and 15 normal tongue tissues. The mRNA expression level was log_2_-transformed to calculate the correlation and fold change with the FPKM value plus 0.01 (to avoid error during log_2_ transformation).

### Cell lines and cell cultures

The CAL-27 cell line was purchased from ATCC (American Type Culture Collection) and TCA-8113 cell line was a gift from Dr. Chen (College of Stomatology, Ninth People's Hospital, Shanghai Jiao Tong University School of Medicine). The cells were maintained in the suggested medium and incubated at 37 °C in a humidified atmosphere of 95% air and 5% CO_2_. All cell lines were STR-authenticated annually by Shanghai Biowing Applied Biotechnology Co. LTD, Shanghai, China.

### Plasmid construction

The overexpression of FZD2 in CAL-27 and TCA-8113 cells was performed using the pCDH-CMV-MCS-EF1-RFP vector (Key Laboratory of Ministry of Education for Gastrointestinal Cancer, School of Basic Medical Sciences, Fujian Medical University, Fuzhou, China). Briefly, a pair of primers was designed and synthesized, as follows: forward, 5'-CGGAATTCGCCACCATGCGGCCCCGCAGCGCC-3' and reverse, 5'-ATAAGAATGCGGCCGCTCACACGGTGGTCTCAC-3'. These primers were used to amplify the complete coding sequence (CDS) of the FZD2 gene (NM_020510) via reverse transcription PCR (RT-PCR). The obtained fragments were digested using the EcoR I-HF and Not I-HF restriction enzymes. The digested fragments were sequenced and then inserted into the pCDH-CMV-MCS-EF1-RFPvector (pCDH-FZD2).

### siRNA transfection

siRNAs targeting FZD2 were designed and synthesized (Gene Pharma, Shanghai, China). The specific sequences of these siRNAs are shown in Table 2. We transfected the siRNAs into CAL-27 and TCA-8113 cells according to the instruction manual for Lipofectamine RNAiMAX (Invitrogen, Catalog # 13778150). Plasmid amplification was accomplished through transformation and pumping. We transfected the cells following the instructions for Lipofectamine 3000 (Invitrogen, Catalog # L3000015).

### Quantitative real-time PCR

The expression of Notch relevant mRNA was measured by quantitative real-time PCR analysis as previously described 4. The sequences of primers for FZD2 are 5'-AGTTCTATCCGCTGGTGAAGGT-3' (forward) and 5'- GCCCAGAAACTTGTAGCTGAGA-3' (reverse), and for ACTB are 5'-CCTGGCACCCAGCACAAT-3' (forward) and 5'-GGGCCGGACTCGTCATACT-3' (reverse). Data were analyzed according to the 2^ -ΔΔCt^ method.

### Western blotting

The protein expression of FZD2 was analyzed by Western blotting as previously described 4 with goat anti-FZD2 antibody (AbCAM, Ab109094, 1:1000 dilution).

### Cell proliferation assay

Cell proliferation was detected using the Cell Counting Kit-8 (CCK8) reagent and the colony formation assay as previously described 4.

### Wound healing assay

The CAL-27 cells that were transfected with siRNA or the pCDH-FZD2 plasmid were seeded in a 6-well plate. The cells grew to monolayers covering the bottom of the plate, at which point 20 μL pipette tips were used to cross-scratch the cells. Then, the medium was replaced at 0 h and 24 h or 36 h postscratching with 0.1% fetal bovine serum DMEM (Dulbecco's Modified Eagle's Medium), and images were taken.

### Cell migration and invasion assay

The cell migration and invasion abilities were measured using 24-well transwell chambers (8-mm pore size) without or with Matrigel, as previously described 4.

### Establish xenograft tumor model

Female BALB/c nude mice that were 6 to 8 weeks of age were purchased from the Center for Animal Experiments of Fujian Medical University. Cells (2×10^6^) were suspended in 0.2 mL serum-free DMEM and injected into the right axillary fossa of each mouse. Tumor size was calculated using the formula V =width^2^× length/2. At the end of the experiment, the tumors were harvested and weighed. The experimental animal protocols were approved by the Animal Care and Use Committee of Fujian Medical University.

### Statistical analysis

The chi-squared test was used to analyze the results of FZD2 immunoreactivity. The results of the other experiments in this study were analyzed by one-way analysis of variance. Nonsignificance (ns) is indicated by P > 0.05, and P < 0.05 was considered statistically significant. *indicates P< 0.05, **indicates P < 0.01.

## Supplementary Material

Supplementary figures and tables.

## Figures and Tables

**Figure 7:**
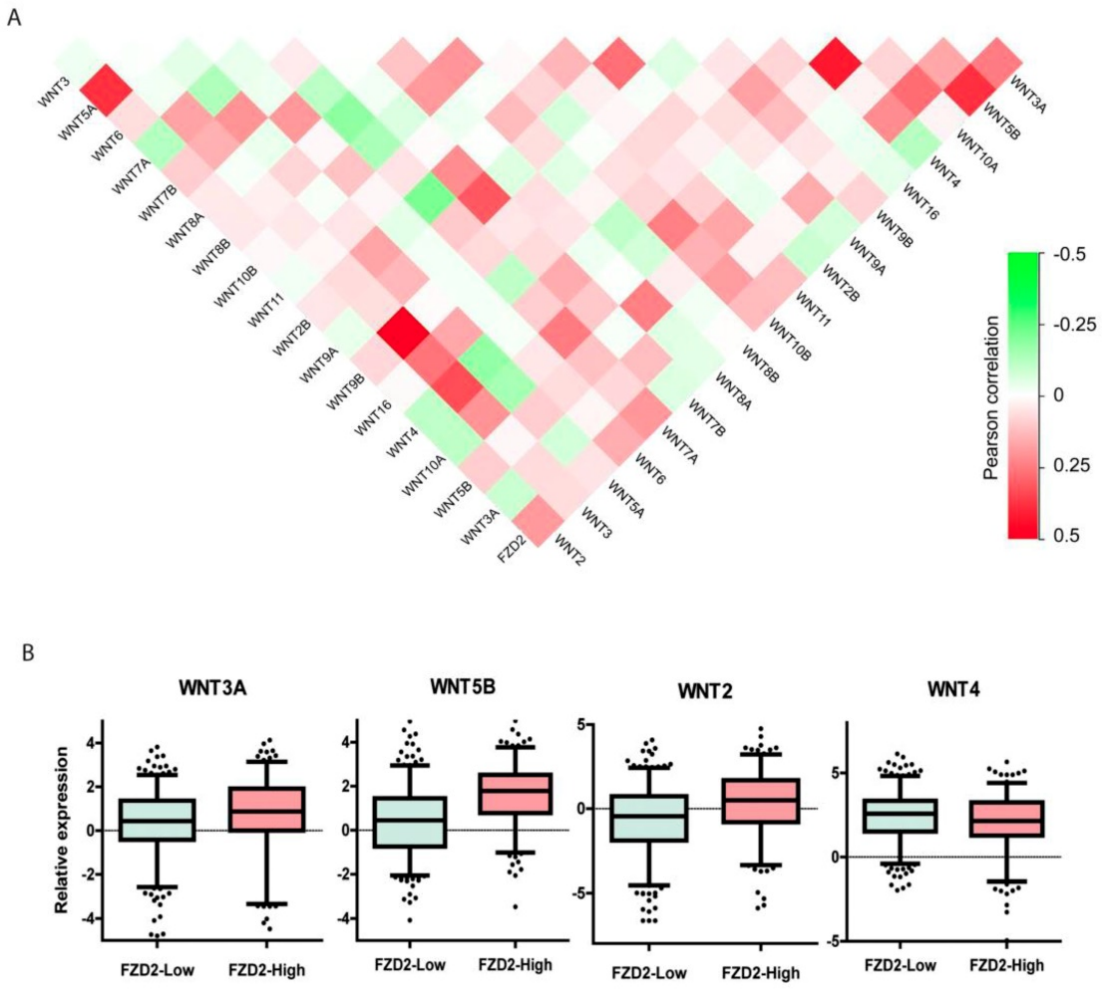
** Coexpression of FZD2 receptor and Wnt ligands in head and neck squamous carcinoma sampl**es. The systematically map of FZD2-Wnt interactions in head and neck squamous carcinoma samples (A, n=501). The expression of WNT3A, WNT5B and WNT2 were significantly increased, while WNT4 was decreased in FZD2-high group (n=194) compared to FZD2-low (n=307) group (B, P<0.01 by t test).

**Table 1:** The expression of FZD2 in tongue cancer samples with different clinical and pathological characters

Characteristics	Cases	Means	SD	P value
Gender				
Female	22	0.8486	0.4731	0.6179
Male	22	0.5023	0.5011	
Age				
Less than 55	20	1.011	0.5162	0.3767
55 and up	24	0.3962	0.4511	
Tumor Stages				
T1 and T2	16	0.6800	0.6040	0.9920
T3 and T4	28	0.6728	0.4197	
Differentiation				
Poorly and Moderately	26	-0.2106	0.5602	0.0291*
Well	18	1.289	0.3937	
Lymph Node metastasis				
N0	26	0.8215	0.4566	0.6131
N1 and N2	18	0.4644	0.5235	

**Table 2:** The sequences of the siRNAs used in the transfection experiments

Name	Sense	Antisense
siRNA-605	5'-GCGAAGCCCUCAUGAACAATT-3'	5'-UUGUUCAUGAGGGCUUCGCTT-3'
siRNA-931	5'-CCCGAUGGUUCCAUGUUCUTT-3'	5'-AGAACAUGGAACCAUCGGGTT-3'
NC	5'-UUCUCCGAACGUGUCACGUTT-3'	5'-ACGUGACACGUUCGGAGAATT-3'
