# Supplementary material for: FZD2 regulates cell proliferation and invasion in tongue squamous cell carcinoma
Source: Int J Biol Sci. 2019 Aug 24;15(11):2330–9. doi: 10.7150/ijbs.33881 (PMC6775310; doi:10.7150/ijbs.33881)
Supplement: Supplementary file 1 — Supplementary figures and tables. [file ijbsv15p2330s1.pdf]

FZD2 regulates cell proliferation and invasion in tongue squamous cell carcinoma

Li Huang et al.

Supplementary figures.

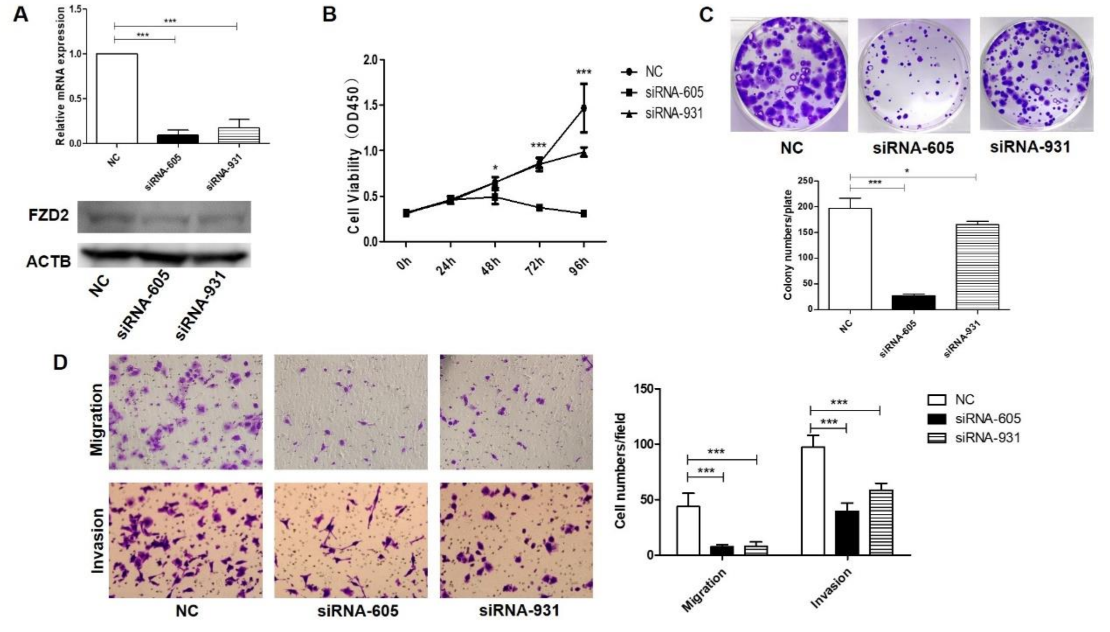


Figure S1. Knockdown of FZD2 inhibited the growth, migration and invasion of TCA-8113 cells in vitro. After siRNA transfection in TCA-8113 cells, the expression of FZD2 was measured by real-time PCR (A, upper panel) and Western blot (A, lower panel) , the cell proliferation was detected by CCK8 assay (B, $P < 0.05$ from 48 h to 96 h) and colony-formation assay (C, $P < 0.005$, n=3). The cells migration and invasion were detected by transwell assay coated with or without matrigel (D, $P < 0.005$), the representative images of transwell inserts coated without (upper panel) or with (lower panel) matrigel.

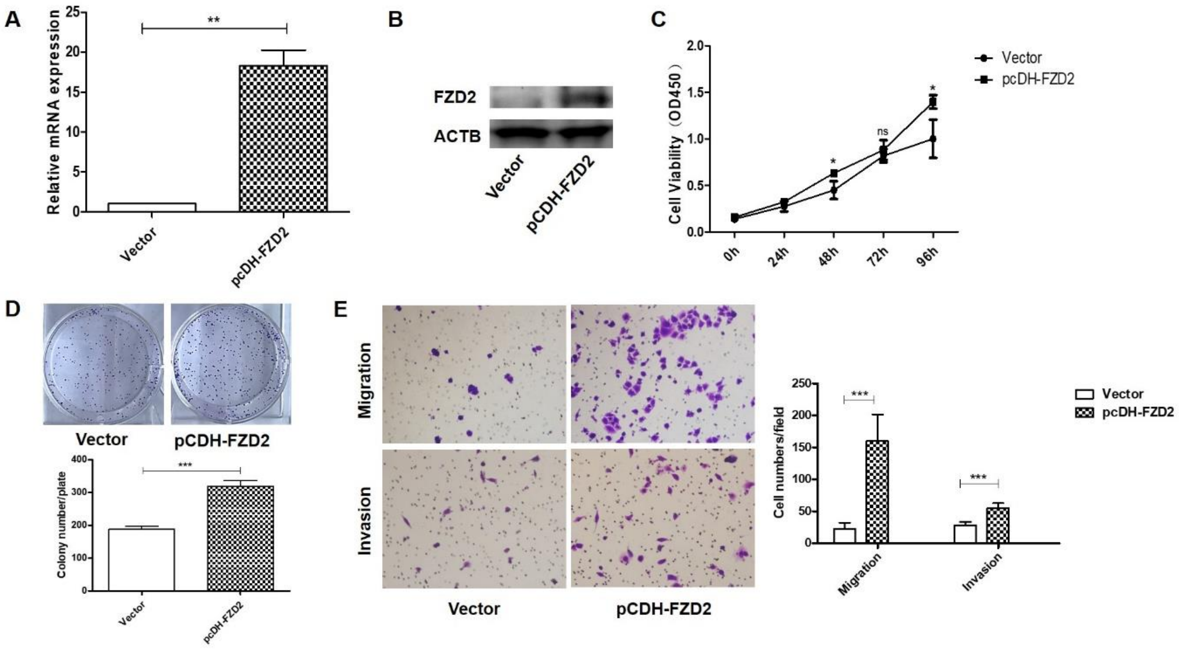


Figure S2. Upregulation of FZD2 in TCA-8113 cells promoted cells growth, migration and invasion in vitro. After transfected with plasmid, the expression of FZD2 was measured by real-time PCR (A) and Western blotting (B), the cell proliferation was detected by CCK8 assay (C, $P < 0.05$ from 48 h to 96 h) and colony-formation assay (D, $P＜0.01$, n=3). The cells migration and invasion were detected by transwell assay coated with or without matrigel (E, $P＜0.005$), the representative images of transwell inserts coated without (upper panel) or with (lower panel) matrigel.
